# Microglial Morphology and Dynamic Behavior Is Regulated by Ionotropic Glutamatergic and GABAergic Neurotransmission

**DOI:** 10.1371/journal.pone.0015973

**Published:** 2011-01-25

**Authors:** Aurora M. Fontainhas, Minhua Wang, Katharine J. Liang, Shan Chen, Pradeep Mettu, Mausam Damani, Robert N. Fariss, Wei Li, Wai T. Wong

**Affiliations:** 1 Unit on Neuron-Glia Interactions in Retinal Disease, National Eye Institute, National Institutes of Health, Bethesda, Maryland, United States of America; 2 Unit on Retinal Neurophysiology, National Eye Institute, Bethesda, Maryland, United States of America; 3 Biological Imaging Core, Office of the Scientific Director, National Eye Institute, National Institutes of Health, Bethesda, Maryland, United States of America; Virginia Commonwealth University, United States of America

## Abstract

**Purpose:**

Microglia represent the primary resident immune cells in the CNS, and have been implicated in the pathology of neurodegenerative diseases. Under basal or “resting” conditions, microglia possess ramified morphologies and exhibit dynamic surveying movements in their processes. Despite the prominence of this phenomenon, the function and regulation of microglial morphology and dynamic behavior are incompletely understood. We investigate here whether and how neurotransmission regulates “resting” microglial morphology and behavior.

**Methods:**

We employed an *ex vivo* mouse retinal explant system in which endogenous neurotransmission and dynamic microglial behavior are present. We utilized live-cell time-lapse confocal imaging to study the morphology and behavior of GFP-labeled retinal microglia in response to neurotransmitter agonists and antagonists. Patch clamp electrophysiology and immunohistochemical localization of glutamate receptors were also used to investigate direct-versus-indirect effects of neurotransmission by microglia.

**Results:**

Retinal microglial morphology and dynamic behavior were not cell-autonomously regulated but are instead modulated by endogenous neurotransmission. Morphological parameters and process motility were differentially regulated by different modes of neurotransmission and were increased by ionotropic glutamatergic neurotransmission and decreased by ionotropic GABAergic neurotransmission. These neurotransmitter influences on retinal microglia were however unlikely to be directly mediated; local applications of neurotransmitters were unable to elicit electrical responses on microglia patch-clamp recordings and ionotropic glutamatergic receptors were not located on microglial cell bodies or processes by immunofluorescent labeling. Instead, these influences were mediated indirectly via extracellular ATP, released in response to glutamatergic neurotransmission through probenecid-sensitive pannexin hemichannels.

**Conclusions:**

Our results demonstrate that neurotransmission plays an endogenous role in regulating the morphology and behavior of “resting” microglia in the retina. These findings illustrate a mode of constitutive signaling between the neural and immune compartments of the CNS through which immune cells may be regulated in concert with levels of neural activity.

## Introduction

Microglia constitute the resident tissue macrophage and primary immune cell of the CNS. Under basal conditions, “resting” microglia demonstrate ramified morphologies and extend fine processes through nearby neural parenchyma in a non-overlapping manner. Although termed “resting”, microglia in the retina and the brain exhibit rapid and extensive dynamism in their processes, enabling each microglial cell to come into close proximity and establish repeated contact with nearby neurons, macroglia, and blood vessels [1], [2], [3]. While a ramified morphology and dynamic process behavior are key phenotypes of “resting” microglia, knowledge on how these are influenced and regulated by extracellular signals is largely incomplete [4]. As a result of their distribution and morphology, microglia are in constant and intimate contact with multiple signals originating from nearby neurons and macroglia. The transformation of a microglial cell from a “resting” state through gradations of “activated” states is thought to depend on the overall balance between “on” signals that promote activation, and “off” signals that repress it [5], [6]. Similarly, the morphology and motility of “resting” microglia are also dynamically influenced by extracellular signals such as ATP and chemokines arising from other surrounding cells [1], [4]. One important and ubiquitous form of intercellular signaling in the brain and retina is neurotransmission. Whether and how microglial morphology and dynamic behavior may be modulated by neurotransmission has not been fully explored.

Recent studies have suggested that “resting” microglial dynamic behavior may play key roles in housekeeping functions such as pruning excess or dysfunctional synapses [7], [8], distributing supportive growth factors to active neurons and astrocytes [9], and regulating synaptic function [10], [11]. These functions are likely to influence neuronal activity in the intact CNS and, in turn, microglia may be expected to be responsive to feedback signals that vary with the ongoing levels of neuronal activity. In conveying information on neuronal activity, neurotransmission may therefore serve as an appropriate mode of communication to microglia to guide their basal morphology and dynamic behavior.

In this study, we address the hypothesis that ionotropic neurotransmission in the CNS serves as a rapid signal that regulates the morphology and dynamism of “resting” microglia. We have employed in our study an *ex vivo* explant preparation of the mouse retina which constitutes a useful model system for addressing neurotransmitter regulation of microglial morphology and behavior. The flat geometry of the retina, unlike that of the larger brain, allows it to be explanted as a thin, intact sheet of optically-accessible neural tissue without the need for tissue slicing. Spontaneous patterns of electrical activity in neurons [12] and macroglia [13], [14], driven and regulated by various forms of endogenous ionotropic neurotransmission, are preserved in flat-mounted retinal explants [15], [16]. Also, retinal microglia in this system display an extensively ramified morphology, a regularly-spaced and non-overlapping distribution [17], [18], as well as marked dynamic behavior [3], [4] that are similar to those documented *in vivo* [19], [20].

Our experimental results using this model system reveal that the specialized ramified morphology of microglia as well as the quantitative dynamics of their motile processes are strongly influenced by retinal neurotransmission and are reciprocally regulated by different modes of neurotransmission. Endogenous ionotropic glutamatergic transmission, specifically that occurring through AMPA and kainate receptors, maintains microglial dendritic morphology in terms of size and complexity and also potentiates process motility. Conversely, ionotropic GABAergic transmission exerts negative regulatory effects on both morphology and motility. These effects do not appear to be mediated by the direct microglial reception of neurotransmission but appear indirectly regulated through a secondary signal in the form of ATP, which is likely released via a mechanism involving pannexin-1 hemichannels. These findings indicate a novel mechanism by which neuronal activity, through regulating ATP release, influences the surveying capacity of microglia by modulating their dendritic size and plasticity. On a more general scale, they also reveal neurotransmission to be an ongoing mode of communication occurring between neuronal and immune compartments in the physiologically active retina.

## Materials and Methods

### Experimental Animals

All animals were handled in strict accordance with good animal practice as defined by the relevant national and/or local animal welfare bodies, and all animal work was approved by the NEI Institutional Animal Care and Use Committee in animal protocols 07-602 and 07-603. Homozygous CX3CR1^GFP/GFP^ transgenic mice [21] on a C57BL/6 background were obtained from The Jackson Laboratory (Bar Harbor, ME). Three to 8 week-old heterozygous CX3CR1^+/GFP^ animals, created by breeding CX3CR1^GFP/GFP^ mice to wild type C57BL/6, mice, were used in live-cell imaging experiments and for immunohistochemical studies. All mice were housed and bred in NIH animal facilities.

### Dynamic Imaging of Microglia in Retinal Explants

CX3CR1^+/GFP^ animals were euthanized and their eyes immediately enucleated and immersed in oxygenated Ringer's solution containing (in mM): 125 NaCl, 5 KCl, 1.5 CaCl_2_, 0.75 MgCl_2_/6 H_2_O, 1.25 NaH_2_PO_4_, 10 D-glucose, 20 HEPES (pH 7.35–7.45). Retinas were dissected free from the eyecup and flat-mounted on black Millipore filter paper (HABP045; Millipore, Billerica, MA) with the ganglion cell layer facing upwards. Flat-mount retinal explants were maintained in Ringer's solution at room temperature in a humidified, oxygenated chamber for no longer than 6 hours after dissection. For imaging experiments, explants were transferred to a stage-mounted, temperature-controlled (32°C) chamber (Bioptechs, Butler, PA) through which oxygenated Ringer's solution was continuously superfused. GFP-labeled microglia were imaged using a confocal microscope (SP2; Leica, Exton, PA) and a 40× (0.80 numerical aperture) water-immersion objective. Multiplane Z-series time-lapse images spanning the dimensions of imaged microglia were collected at a 512×512 pixel resolution at a rate of one image stack every 10 seconds. Agonists and antagonists were administered by superfusion into the recording chamber. Microglial morphology and motility were evaluated before, during, and after superfusion of each agent. The duration of a typical recording was approximately 25–33 minutes (150–200 image stacks). Neurotransmitter agonists evaluated were: AMPA, kainate, NMDA (all from Tocris), glutamate, GABA, and ATP (all from Sigma). Antagonists evaluated were: NBQX (1,2,3,4-tetrahydro-6-nitro-2,3-dioxo-benzo[f]quinoxaline-7-sulfonamide disodium) a kainate/AMPA receptor antagonist; GYKI-52466, an AMPA receptor antagonist; APV (2-amino-5-phosphonopentanoic acid), an NMDA receptor antagonist; bicuculline, a ionotropic GABA_A_ receptor antagonist; suramin, a broad-spectrum P2 receptor antagonist (all from Tocris Bioscience, Ellisville, MO). Apyrase (Sigma, St. Louis, MO), an enzyme catalyzing the hydrolysis of ATP, was also evaluated.

### Image Analysis

Image processing was performed using NIH ImageJ software. Maximum intensity projections of z-series stacks were created and aligned in the x–y plane to create 2-dimensional time-lapse movies. Quantitative analysis of microglia processes were limited to those fully contained in the imaging space, avoiding focus change artifacts that might have been mistaken for structural changes. For morphological analysis, the average of 3 consecutive time-series images at a particular phase in the recording was obtained. Dendritic tree area as a morphological parameter was determined by circumscribing the area outlined by the ends of dendritic processes using a smooth polygon tool in NIH ImageJ. In addition, the maximum intensity projection image was binarized and a topological skeleton derived from the binary image using the “skeletonize” function in ImageJ. Total dendritic length and total branch point number were calculated from the skeletonized image. Quantitative comparison of mean process motility was calculated for individual cells; for each cell, sequential images in a time-series (taken 10 s apart) were aligned and binarized, and then image subtractions between consecutive images were performed. The area of summed pixels in subtracted images (area of change in pixels added or lost) was used as a measure of the instantaneous rate of structural change in the motile processes. An average rate for a particular segment of the recording was then calculated as the mean of instantaneous rates across that segment. In this way, mean process motilities in different recording segments corresponding to the presence or absence of an agonist or antagonist were then quantified and compared.

### Electrophysiological recordings

Retina explants flat-mounted onto filter paper were sectioned into 150 µm-thick retinal sections using a spring loaded blade. Retinal sections were placed into a stage-mounted recording chamber and superfused (rate of 0.5–1 ml/min) with a solution that contained (in mM): 115 NaCl, 3.1 KCl, 2.28 MgSO4, 2.0 CaCl2, 6 glucose, 1 succinate, 1 lactate, 1 malate, 1 pyruvate, 3.25 NaHCO (pH 7.4 with 5% CO2). Whole-cell recordings were made of GFP-positive microglia cells with somata in the inner plexiform layer or the ganglion cell layer. Recording electrodes (5∼10 MΩ) contained (in mM): 80 KCl, 30 CsCl, 2 MgSO4, 20 HEPES, 10 EGTA, 5 ATP, 0.5 GTP (pH 7.4). Recordings were obtained with an Axopatch 200B amplifier (Molecular Devices, Sunnyvale, CA), and currents low–pass filtered at 5 kHz using the 4 pole Bessel filter on the amplifier. Data was digitized at 10 kHz using an ITC-18 interface (Instrutech, Bellmore, New York) controlled by a Dell computer running IgorPro 6.0 (WaveMetrics, Portland, Oregon). Data was analyzed with IgorPro 6.0. To evaluate responses of microglia cells to receptor agonists, a glass pipette (2–3 µm tip) connected with a custom-made pneumatic pump was used for local application of agonists (3–5 psi for 1 s). The following agonists were applied (in mM): 10 ATP, 1or 10 Glutamate, 100 AMPA, and 1 GABA.

### Immunofluorescence studies

Enucleated eyes were opened at the limbus, and fixed in 4% paraformaldehyde in 1× PBS (pH 7.3) for 30–45 minutes. Retinas were then detached, rinsed in PBS, and incubated in ICC buffer (1× PBS, 0.5%BSA, 0.2% Tween 20, 0.05% sodium azide, pH 7.3) containing 5% normal goat serum for 4 hours at room temperature to decrease non-specific binding. Primary polyclonal antibodies to GluR2/3 and GluR4 (1∶50 dilution, Millipore) in ICC buffer were then added and incubated with the tissue for 5 days at 4°C. Retinas were rinsed with ICC buffer and incubated with secondary antibody (conjugated with Alexa Fluor-555, 1∶500 dilution, Molecular Probes) for 4 hours at room temperature.

### Colocalization analysis

Images of a single confocal scan were analyzed with custom software (kindly provided by Dr. Brady Trexler) that quantifies the average spatial overlap between two types of cellular structures labeled in different spectral channels [22]. Two-dimensional analysis image sub-fields (23×23 pixels) were placed symmetrically around areas of punctate labeling of glutamate receptors (labeled in red) adjacent to microglia processes, cropped from the image, aligned spatially, and then averaged to generate a plot of intensity versus pixel positions (using IgorPro 6.0). Intensity plots were generated with signals in the red channel (distribution of glutamate receptor labeling) and also in the green channel (distribution of GFP-labeled microglial processes). Overlap of signals between the two channels indicated colocalization of the glutamate receptors with microglial processes, whereas the presence of a caldera-like plot in one channel and a converse peak from the other channel indicated an anti-correlation, i.e., a spatially-exclusive relationship and the absence of colocalization.

### Statistical Analysis

Statistical analyses were performed using computer software (GraphPad Prism, La Jolla, CA). The effect of agonists or antagonists compared to baseline was assessed statistically using a paired, two-tailed t-test. Data were expressed as mean ± SEM. Significance was considered to be achieved at P<0.05.

## Results

### Ionotropic glutamatergic transmission positively regulates resting microglial dendritic morphology and process motility

**Figure 1:**
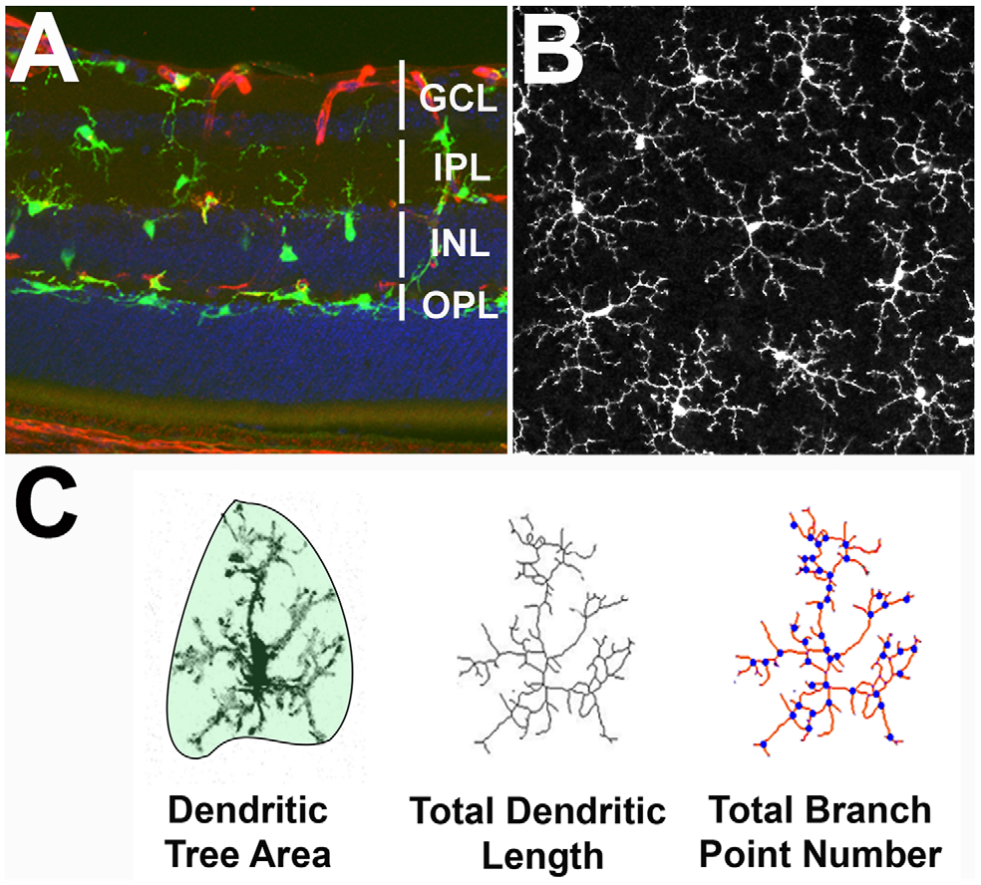
Dendritic Morphology of Retinal Microglia. ***A***. Retinal section from an adult mouse (2–3 months of age) showing the laminar distribution of retinal microglia (*green*) in the inner half of the retina, including the ganglion cell layer (GCL), inner plexiform layer (IPL), inner nuclear layer (INL), and outer plexiform layer (OPL). ***B***. Dendritic arbors of individual retinal microglia, as seen in a confocal image of a flat-mounted retinal explant captured in the horizontal plane of the retina, have symmetrically oriented processes that do not overlap with those in neighboring cells. ***C***. Analysis of the dendritic morphology of microglia in *ex vivo* retinal explants from 2-dimensional image projections of confocal images. Three morphological parameters were analyzed: 1) *Dendritic tree area* (area circumscribed by the polygonal object defined by connecting the outer points of the dendritic ramified arbor, *shadowed*), 2) *Total dendritic length* (sum of all dendritic segments identified in a skeletonization of the arbor), and 3) *Total branch point number* (sum of branch points identified in a skeletonized rendition of the arbor, *points in blue*).

We demonstrated in previous studies that ramified “resting” microglia in the retina, like those elsewhere in the CNS, display rapid surveying movements in their cellular processes while maintaining invariant soma positions and homeostasis in the overall size and symmetry of their ramified dendritic arbors [3], [4], [23]. These dendritic arbors show non-overlapping territories and lack polarization in any preferential horizontal direction under resting conditions (Fig 1 A, B). We used time-lapse confocal imaging to follow the structure and dynamic behavior of GFP-labeled microglia in *ex vivo* retinal explants. Recordings were conducted in the presence of antagonists and agonists to ionotropic neurotransmission in the recording bath solution. We employed three separate parameters to assess microglial dendritic morphology (Fig. 1C): 1) dendritic tree area, defined as the 2-dimensional area formed by connecting the outermost points of a cell's dendritic tree and approximating the zone occupied by the cell's dynamic surveying processes; 2) total dendritic length, defined as the sum of all the lengths of the cell's processes; and 3) total number of branch points in the dendritic tree, reflecting the overall dendritic complexity of the cell.

**Figure 2:**
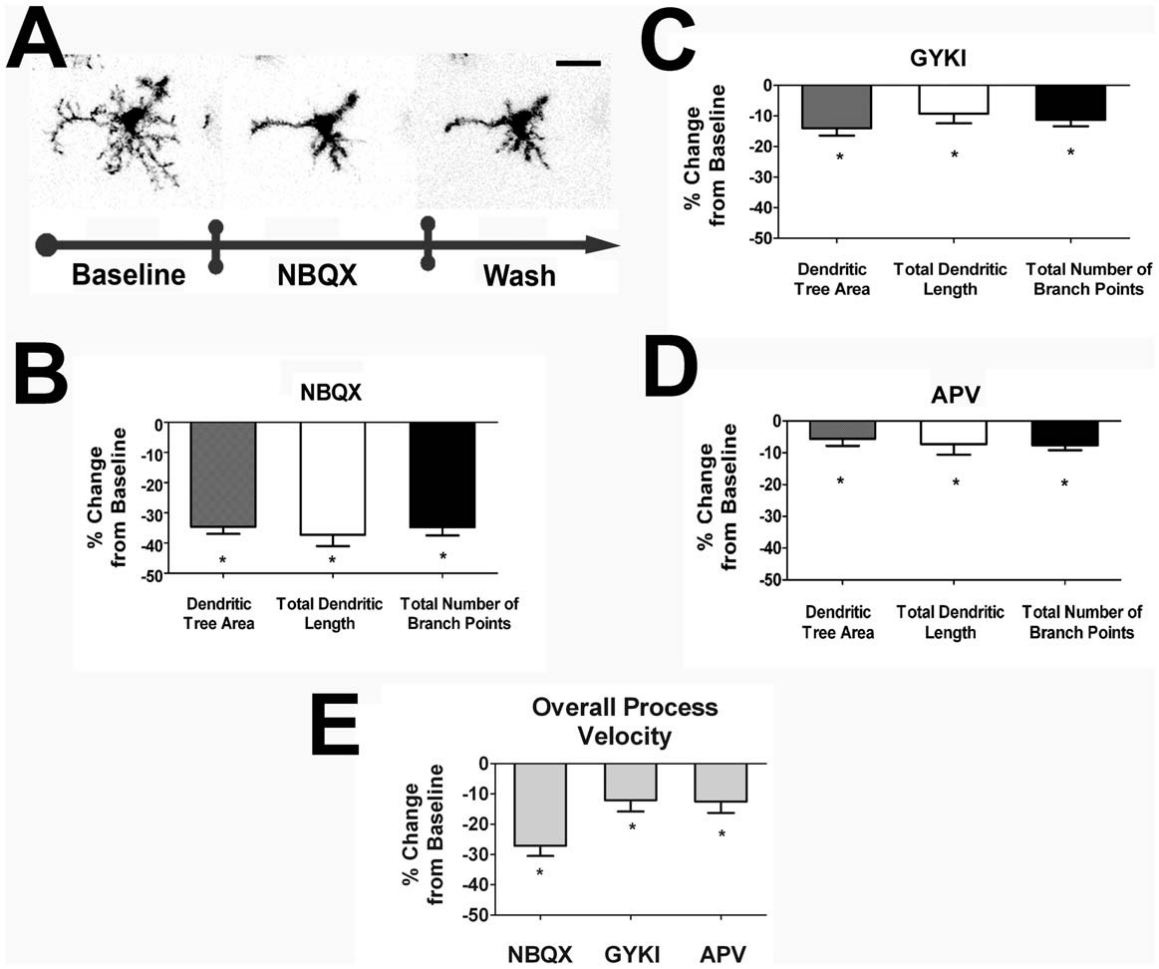
Endogenous ionotropic glutamatergic neurotransmission positively regulate dendritic morphology and process dynamics of retinal microglia. ***A***. Confocal images of a representative retinal microglia cell under control conditions (*left*), in the presence of NBQX (10µM), a glutamate receptor antagonist of AMPA- and kainate-gated channels (*middle*), and during washout of NBQX (*right*). Microglial dendritic morphology is significantly reduced in size and complexity in the presence of ionotropic glutamatergic blockade with NBQX, and is partially reversed with washout with Ringer's solution. Scale bar = 20µm. ***B,C*** Quantification of morphological parameters demonstrate that all three morphological parameters are significantly decreased from baseline values in the presence of NBQX (10µM; n = 42 cells), as well as GYKI-52466 (100 µM; n = 48 cells), an antagonist of AMPA-gated channels. ***D***. Similar recordings were also performed with the application of APV (100 µM; n = 29 cells), an antagonist of ionotropic NMDA-gated glutamatergic channels. All 3 morphological parameters were also significantly reduced, albeit to a smaller extent than seen with NBQX. ***E***. Overall process velocities were also calculated from the above recordings, with NBQX (n = 44 cells), GYKI (n = 52 cells) and APV (n = 26 cells) applications significantly decreasing baseline process motility, with NBQX exerting a larger decrement compared to GYKI or APV. All significant changes (p<0.05) from baseline are indicated by an asterisk (*).

We first examined the role of endogenous excitatory ionotropic glutamatergic transmission on microglia morphology and dynamism. Application of NBQX (10 µM), a blocker of endogenous glutamatergic signaling through AMPA and kainate receptors, induced rapid and progressive decrease in the size and complexity of the ramified dendritic arbor (Fig. 2A). Changes in morphology were evident 1–3 minutes after the initiation of ionotropic glutamatergic receptor blockade and were sustained throughout the period of blockade (Movie S1). No changes in soma position were noted however, and no overt cellular migration was documented. Transition to an amoeboid morphology, suggestive of a typical “activated” state, was not observed. The absence of overt microglial activation was also indicated by the partial recovery of overall dendritic structure 7–8 minutes following antagonist wash-out with superfused oxygenated Ringer's solution. Upon NBQX blockade, all three morphological parameters of dendritic tree area, total dendritic length, and total branch point number decreased to ∼60–70% of their baseline values (dendritic tree area: −34.6%, total dendritic length: −37.3%, and total branch points: −34.8%) (Fig 2B). GYKI-52466 (100 µM), an AMPA receptor antagonist, in similar experiments induced significant, but relatively smaller decreases in the 3 morphological parameters to ∼85–90% of baseline values (dendritic tree area: −14.0%, total dendritic length: −9.3%, and total branch points: −11.3%) (Fig 2C). . Blockade of NMDA receptor-mediated glutamatergic transmission using APV (100 µM), a selective NMDA receptor antagonist, induced small (∼5–8% decrease) but significant decreases in morphological parameters, indicating a smaller contribution of endogenous NMDA-mediated neurotransmission to maintaining dendritic morphology (Fig 2D). The effect of antagonists, NBQX, GYKI, and APV, were also evaluated with respect to the ability to influence the average velocity of motile “surveying” microglial processes. All 3 antagonists induced significant decreases in process dynamism, with NBQX inducing the largest decrease in motility (NBQX: −27.1%, GYKI: −12.1%, and APV: −12.5%) (Fig 2E). These results indicate that endogenous ionotropic glutamatergic transmission occurring through both AMPA and kainate receptors, and, to a smaller extent, NMDA receptors, positively regulate microglial dendritic structure and process dynamism.

**Figure 3:**
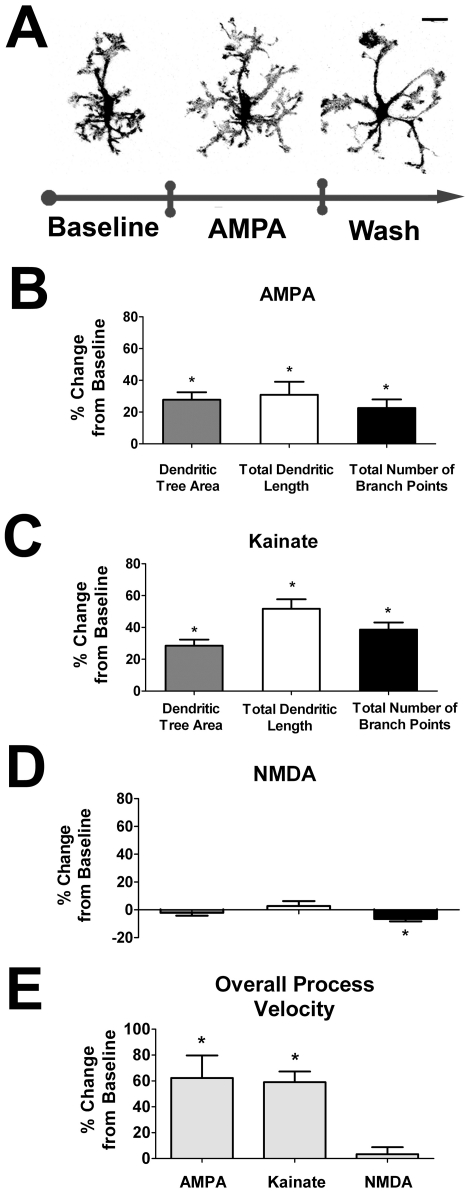
Exogenous ionotropic glutamatergic neurotransmission increases retinal microglia dendritic morphology and process dynamics. ***A***. Confocal images of a representative retinal microglia cell *ex vivo* under control conditions (*left*), with the bath application of AMPA (100 µM)(*middle*), and during washout (*right*). Microglial dendritic morphology is significantly increased in size and complexity during stimulation with AMPA with partial recovery during washout. Scale bar = 20µm. ***B–C***. Quantification of morphological parameters demonstrate that all three morphological parameters of dendritic tree area, total dendritic length, and total number of branch points are significantly increased in response to AMPA (100 µM; n = 33 cells) and kainate (100 µM; n = 42 cells) application. ***D***. Microglia morphology however was relatively stable with the application of NMDA (100 µM; n = 54 cells) in the presence of glycine (10 µM). ***E***. Overall, process velocities were increased in response to AMPA and kainate, but remained unchanged when NMDA was applied. All significant changes (p<0.05) from baseline are indicated by an asterisk (*).

To assess the effect of exogenous ionotropic glutamatergic application on retinal microglia, we exposed retinal explants to AMPA (100 µM in Ringer's solution) by bath application through the perfusion superfusate at a defined point during live-cell recordings. Microglia were observed to respond rapidly 1–3 minutes after application with increases in dendritic structure, dendritic field size, and complexity (Fig 3A and Movie S2), with all three morphological parameters increasing by 22–31% above baseline values (Fig. 3B). Application of kainate (100µM) in analogous recordings produced similar effects, with morphological parameters increasing by 28–52% (Fig. 3C). In contrast, NMDA (100µM, in the presence of 10µM glycine) had little effect on overall dendritic morphology (Fig. 3D). The overall process velocity of “surveying” processes also increased with applications of AMPA (+62.3%) and kainate (+59.1%), but was unchanged with NMDA (Fig. 3E). Interestingly, application of glutamate (1mM), which serves as an agonist on both ionotropic and metabotropic glutamate receptors, did not result in either significant changes on any of the 3 dendritic morphological parameters or on overall process motility (data not shown).

### Resting microglial dendritic morphology and process motility are negatively regulated by ionotropic GABAergic transmission

**Figure 4:**
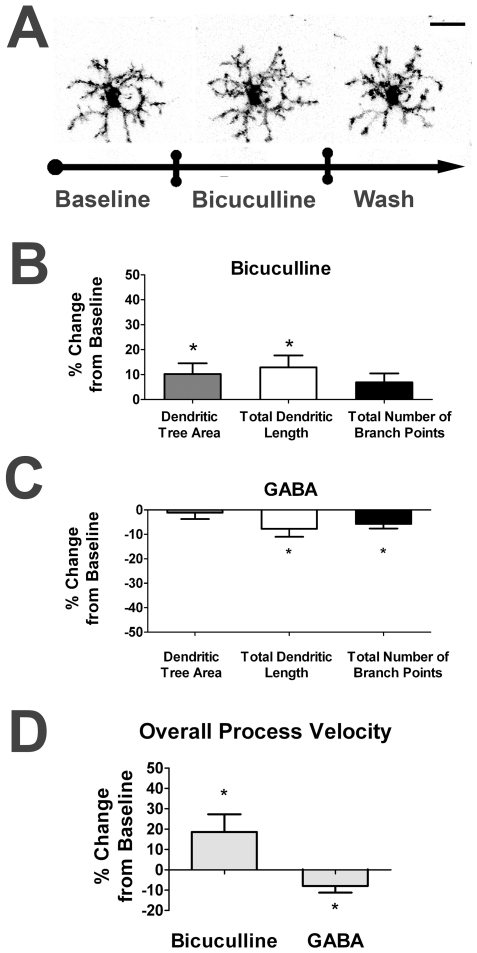
Ionotropic GABAergic neurotransmission decreases retinal microglia dendritic morphology and process dynamics. ***A***. Confocal images of a representative retinal microglia cell under control conditions (*left*), in the presence of bicuculline (150 µM)(*middle*), an antagonist of ionotropic GABA_A_ receptors, and during washout (*right*). Microglial dendritic morphology is slightly increased in size during stimulation with bicuculline, with partial recovery during washout. Scale bar = 20µm. ***B***. Dendritic tree area and total dendritic length was slightly but significantly increased in the presence of bicuculline, while branch point number was unchanged (n = 53 cells). ***C***. Conversely, application of GABA (1mM; n = 23 cells) exerted slight increases in total dendritic length and branch point number, but did not change dendritic tree area significantly. ***D***. Overall process velocity was increased with GABAergic blockade with bicuculline and decreased with GABA application. All significant changes (p<0.05) are indicated by an asterisk (*).

We next examined the role of inhibitory ionotropic GABAergic transmission on microglial morphology and dynamism. To evaluate the role of endogenous GABAergic neurotransmission, we applied bicuculline (150µM), an antagonist to ionotropic GABA_A_ receptors during *ex vivo* microglial imaging. In contrast to ionotropic glutamatergic blockade, we observed an increase in dendritic structure and dynamism after blockade of GABA_A_-receptor mediated neurotransmission (Fig. 4A and Movie S3). All three morphological parameters increased (Fig. 4B), but to a lower extent (∼7–13% increase over baseline values) than that seen with AMPA or kainate application. Conversely, bath application of GABA (1mM) induced relatively small but significant decreases in 2 out of 3 morphological parameters; total dendritic length and total branch point number decreased (Fig. 4C) while dendritic tree area remained relatively unchanged. In terms of overall process velocity, blockade of GABAergic transmission with bicuculline moderately increased process velocity (+18.6%), while exogenous GABA application decreased it slightly but significantly (−7.9%) (Fig. 4D). In either case, no migratory movements of microglia cell soma were observed. These results indicate that ionotropic GABAergic transmission in the retina negatively regulates microglial morphology and dynamic behavior but may be less influential compared to ionotropic glutamatergic transmission as mediated by AMPA- and kainate receptors.

### Microglial dendritic morphology and process motility are positively regulated by extracellular ATP

**Figure 5:**
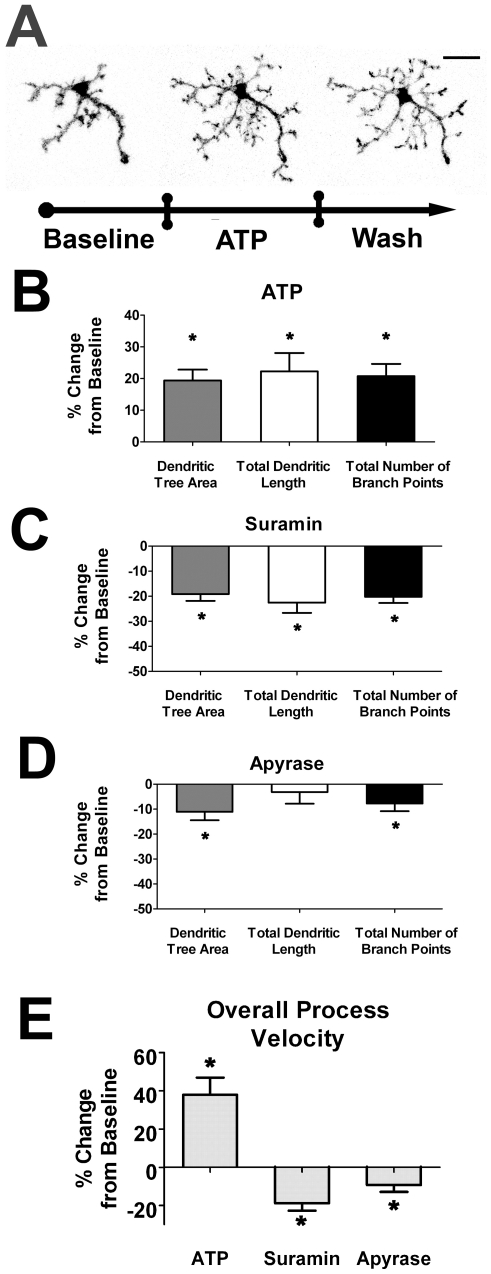
Extracellular ATP increases dendritic morphology and process dynamics of retinal microglia. ***A***. Confocal images of a representative retinal microglia cell under control conditions (*left*), with the bath application of ATP (1mM) (*middle*), and during washout (*right*). Microglial dendritic morphology is significantly increased in size and complexity with ATP. Scale bar = 20µm. ***B***. All morphological parameters were significantly increased in the presence of ATP, (n = 58 cells). ***C–D***. Conversely, morphological parameters were decreased in the presence of suramin (100 µM; n = 25 cells), broad-spectrum antagonist of P2 receptors, and also, to a lesser extent, with apyrase (10U/ml; n = 38 cells), which catalyzes the hydrolysis of extracellular ATP. **E**. Overall process velocity was increased with ATP application and decreased in the presence of suramin and apyrase. All significant changes (p<0.05) from baseline are indicated by an asterisk (*).

Previous studies have demonstrated that cultured microglia *in vitro* can be induced by extracellular ATP to undergo membrane ruffling and exhibit increased chemokinesis [24]. When applied in a gradient or locally, ATP can also induce microglia whole-cell chemotaxis, and attract microglial processes [1], [24], likely by acting through P2X- and P2Y- receptors [23], [25], [26]. Consistent with these previous observations, our recordings in the retina also demonstrated that bath application of ATP (1mM) induced a rapid increase in the size and complexity of dendritic structure (Fig 5A,B, and Movie S4), increasing all three morphological parameters significantly. Application of suramin (100µM), a broad spectrum P2 receptor antagonist, also exerted a general decrease in morphological parameters (Fig. 5C). Application of apyrase (10U/ml), an enzyme that catalyzes the hydrolysis of extracellular ATP, also decreased morphological parameters, but to a lesser extent (Fig. 5D). In terms of process velocity, ATP induced increases in this parameter while suramin and apyrase decreased it (Fig. 5E). As with ionotropic neurotransmission, neither upregulation nor blockade of purinergic signaling induced overt cellular migration over the time-scale of these recordings (∼8 minutes).

### Resting microglia cells in the retina respond directly to exogenous ATP but not glutamatergic or GABA agonists

**Figure 6:**
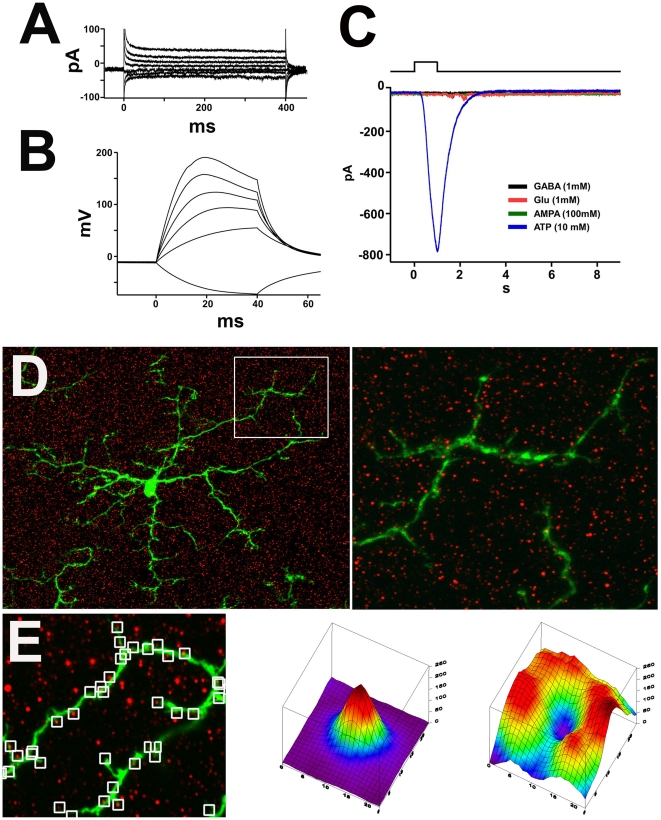
Resting retinal microglia lack direct responses to glutamatergic or GABAergic agonists. **A**. Representative whole cell recordings (n = 8) from green fluorescent protein (GFP)-positive microglia in retinal slices from CX3CR1^+/GFP^ transgenic mice. Under voltage clamp configuration, cells were held at −70 mV and a series of voltage steps (400 ms, 20 mV steps from −40 mV) were applied. Microglia cells did not display delayed rectified K+ currents in response that would indicate an active state. ***B***. Under current clamp configuration, a series of currents (40 ms; −20, 20, 40, 60, 80 pA) were injected into cells. The large membrane potential responses reflect the high input resistance of microglia cells. ***C***. Representative traces showing whole cell currents in retinal microglia. Microglia were voltage clamped at −20 mV, near the resting membrane potential. Local application of ATP by pressurized puff (1 s, 10 mM, n = 4) elicited a large inward current that rose and decayed quickly over several seconds. GABA (1mM, n = 4), Glutamate (1mM, n = 4), or AMPA (100 mM, n = 3), failed to elicit any response. ***D***. Immunolabelling of AMPA receptors, GluR2 and GluR3 (red) in the retina of a CX3CR1^+/GFP^ mouse. The distribution of GluR2 and GluR3 are visible as discrete puncta in the inner plexiform layer (*left*) that are located around microglial processes (*green*) but are not visibly colocalized on microglial processes (expanded inset at higher magnification, *right*). ***E***. Quantitative analysis of colocalization demonstrates that GluR2/3-positive puncta were not located on microglial processes. Multiple square insets (measuring 23 by 23 pixels) were sampled from the image; each inset was centered on a single GluR2/3-positive punctum in the vicinity of the microglial dendritic process (*left*). An averaged plot of intensity versus pixel position was plotted for the red channel (GluR2/3-localization, *middle*) and the green channel (microglial GFP-localization, *right*). The peak in the red channel (middle) reflects the discrete puncta nature of the Glu2/3 distribution, while the caldera-like pattern in the green channel indicates that the location of microglial processes are anti-correlated with that of GluR2/3, signifying that these AMPA receptors are not colocalized with microglial processes.

The regulation of microglial responses to ionotropic glutamatergic and GABAergic signaling may potentially be directly mediated via receptors on the surface of microglia [27], [28], [29]. Alternatively, it may be mediated indirectly via the release of secondary signals which are then received by microglia. To explore these possibilities, we examined direct electrophysiological responses of microglia to neurotransmitters by performing whole cell patch clamp recordings in GFP-labeled ramified microglia in freshly prepared retinal slices. Under voltage clamp configuration, cells were held at −70 mV and a series of voltage steps (400 ms, 20 mV steps from −40 mV) were applied. Retinal microglia were found to be electrically quiescent under these conditions (Fig. 6A). No delayed outward rectifying K+ currents were noted, indicating that microglia were not in an “activated” state [30], [31]. Under a current clamp configuration, a series of currents (40 ms; −20, 20, 40, 60, 80 pA) were injected into patched microglia. Proportional increases in membrane potential were induced but no action potential firing patterns were elicited (Fig. 6B). To assess microglial responses to direct neurotransmitter effects, microglia cells were voltage-clamped at −50mV, and agonists including glutamate, AMPA, GABA or ATP were applied directly onto patched cells using a pneumatic picospritzer. Direct applications of glutamate, AMPA, or GABA all individually failed to elicit any detectable inward or outward currents in microglia. In contrast, local application of ATP produced a large inward current (Fig. 6C). These results indicate that retinal microglia cells respond directly to ATP but not to glutamatergic or GABAergic agonists.

We also performed immunohistochemical localization of AMPA-type glutamate receptor subunits in the retinal explants using antibodies to GluR2/3 and GluR4. The studies were performed on retinal tissue from CX3CR1^+/GFP^ transgenic animals, enabling us to assess whether the pattern of glutamatergic receptor expression was colocalized with the GFP-labeled retinal microglia. Confocal microscopic analysis demonstrated the presence of GluR2/3 and GluR4 receptor units as discrete puncta in the inner plexiform and outer plexiform layers, but these could not be colocalized on the surface of microglial cells in rotational views (Fig 6D). Additionally, image analysis of glutamate receptor puncta in the vicinity of microglia processes also demonstrated a lack of colocalization with GFP-positive microglial processes (Fig. 6E). The lack of direct microglial electrophysiological responses to neurotransmitter application, together with the absence of relevant receptors on the surface of microglia, indicate that glutamatergic- and GABAergic-mediated regulation of microglial morphology and behavior may be indirectly mediated, with ATP acting as an intermediate signal.

### ATP-mediated regulation of microglial morphology and dynamic behavior is dominant over regulation by ionotropic glutamatergic transmission

**Figure 7:**
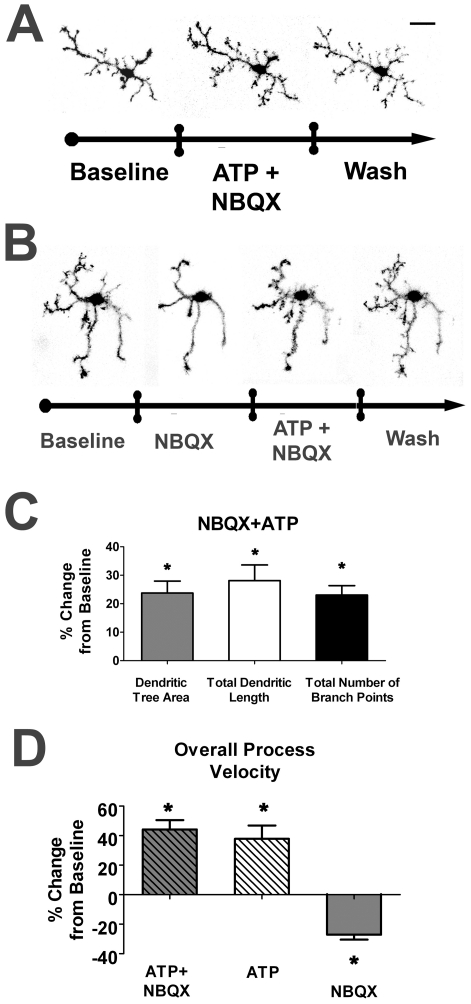
ATP Application Rescues Microglial Morphology and Motility from the Negative Effects of Glutamatergic Blockade. ***A***. Confocal images of a retinal microglial cell *ex vivo* under control conditions (*left*), in the presence of NBQX (10µM) and ATP (1mM) (*middle*), and during washout with Ringer's solution (*right*), demonstrating a net increase of dendritic structure, as is typically seen with ATP alone. ***B***. Similar images showing the effect of sequential addition of NBQX (10µM) alone, followed by a combination of NBQX (10µM) and ATP (1mM), demonstrating that the loss of dendritic structure induced glutamatergic blockade can be reversed by concurrent application of ATP. ***C***. Morphologic parameters of dendritic tree area, total dendritic length, and branch point number, were all increased with concurrent NBQX (10µM) and ATP (1mM) application. (n = 36 cells) ***D***. Overall process velocity was also increased with concurrent NBQX (10µM) and ATP (1mM) application, to a similar degree seen with ATP alone (1mM), and in the opposite direction to the changes induced with the application of NBQX alone (10µM). All significant changes (p<0.05) from baseline are indicated by an asterisk (*).

As both ATP and ionotropic glutamatergic transmissions feature prominently in the regulation of morphology and motility in retinal microglia, we examined the interaction between ATP and glutamatergic signaling. We performed recordings in which ATP was applied concurrently with glutamatergic blockade with NBQX. Unlike the reduction of microglial dendritic morphology seen with the application of NBQX alone, the concurrent application of both ATP and NBQX failed to decrease dendritic morphology. Instead, microglial dendritic morphology increased, similar to that seen with the application of ATP alone (Fig. 7A, Movie S5). Sequential applications of NBQX, followed by NBQX and ATP together (Fig. 7B, Movie S6) demonstrates that ATP is able to overcome the negative effect of NBQX on microglial morphology and motility. Quantification of morphology parameters showed that all three parameters increased significantly (∼23–29% increase) over baseline values when NBQX and ATP were applied together, similar to when ATP was applied alone (Fig. 7C). In terms of overall process velocity, the positive effect of ATP and NBQX combined were again similar to that seen when ATP was applied alone, and opposite to that seen when NBQX was applied alone (Fig. 7D). These results suggest that ATP effects may occur “downstream” of those mediated by ionotropic glutamatergic transmission.

**Figure 8:**
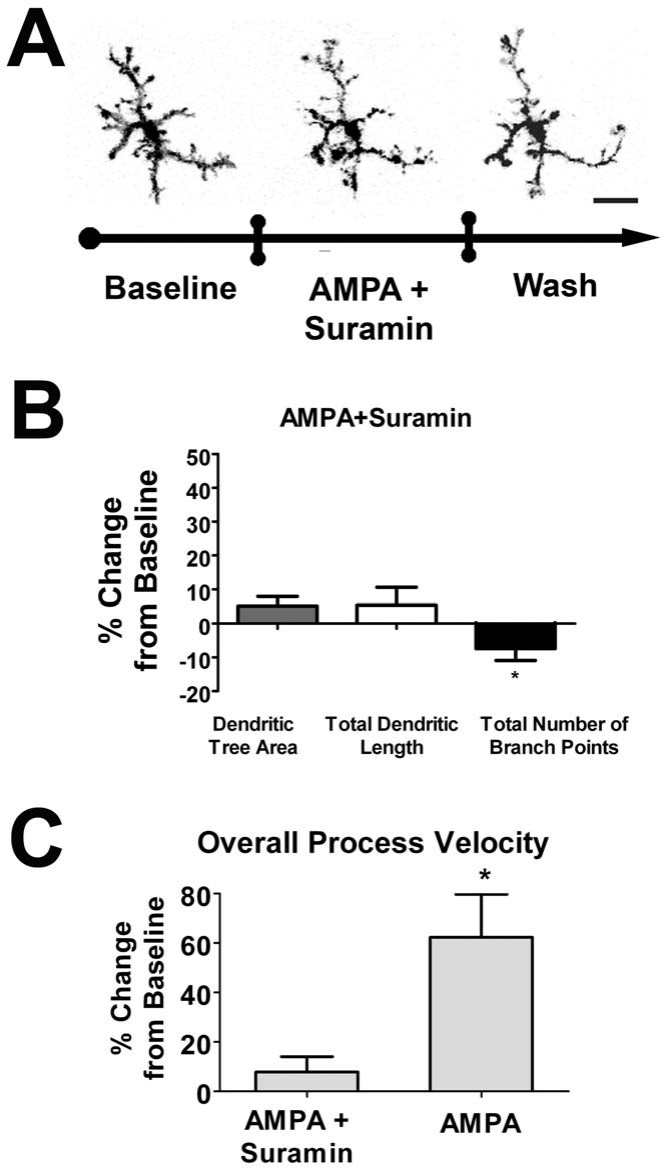
P2 receptor blockade by suramin neutralizes the positive effects of exogenous AMPA application on microglial morphology and process motility. ***A***. Confocal images of a retinal microglial cell *ex vivo* under control conditions (*left*), in the presence of AMPA (1mM) and Suramin (100µM) (*middle*), and during washout with ringers (*right*), demonstrating that the marked increase in microglia dendritic size and complexity is neutralized in the presence of suramin. ***B***. Morphologic parameters of dendritic tree area and total dendritic length were unchanged, and branch point number slightly decreased with concurrent AMPA (1mM) and Suramin (100µM) application; (n = 56 cells). ***C***. Overall process velocity was unchanged with concurrent AMPA (1mM) and Suramin (100µM) application, as compared to the increase induced with the application of AMPA alone (1mM). All significant changes (p<0.05) from baseline are indicated by an asterisk (*).

Similar imaging experiments were also performed with the concurrent blockade of P2 receptors with suramin and the concurrent application of AMPA. The increase of microglial morphology parameters typically seen in response to AMPA application was significantly blunted by the concurrent blockade of P2 receptors (Fig. 8A), and none of the three morphological parameters was significantly increased above baseline, and one of three (total branch point number) was slightly and significantly decreased (Fig. 8B). Similarly, overall process velocity was not significantly affected by AMPA when suramin was concurrently applied (Fig. 8C). The ability of P2 blockade to prevent AMPA-induced increases in morphology and dynamic behavior are consistent with the notion that AMPA-mediated effects on microglia are indirect and involve ATP as an intermediary signal.

### ATP release relevant to the regulation of microglial morphology and dynamic behavior involves pannexin-1 hemichannels

**Figure 9:**
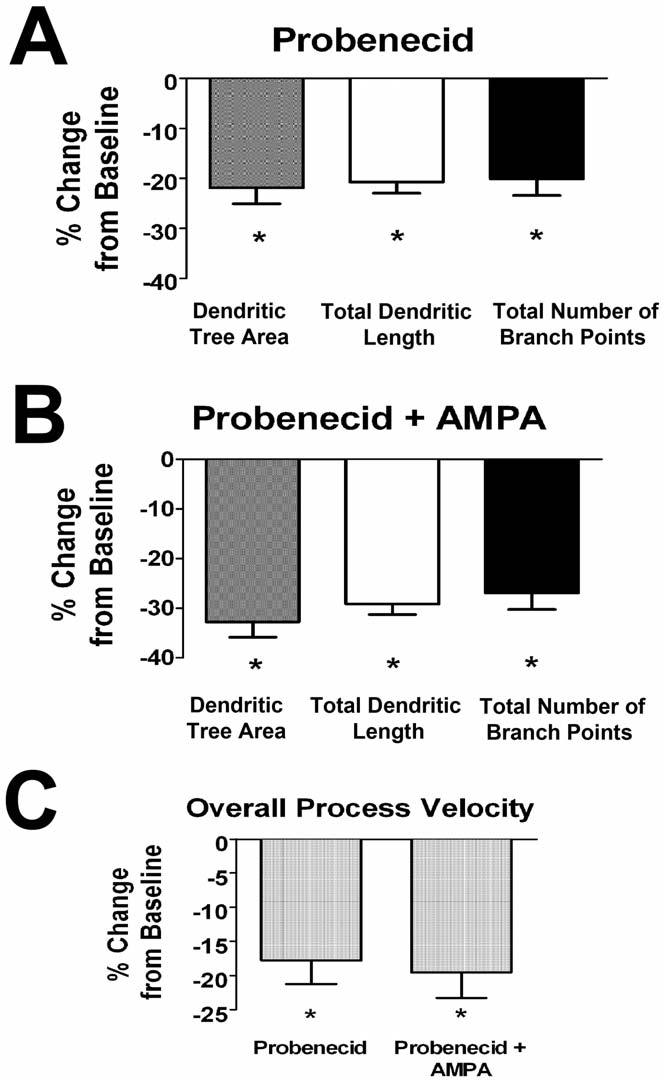
Pannexin-1 hemichannel blockade by probenecid decreases dendritic morphology and process dynamics of retinal microglia. ***A***. Microglial morphologic parameters of dendritic tree area, total dendritic length, and branch point number were significantly decreased by the application of probenecid (1mM), an antagonist of pannexin-1 hemichannels; (n = 46 cells). ***B***. Morphological parameters were similarly decreased with concurrent AMPA (1 mM) and probenecid (5mM) application, indicating that AMPA-induced ATP_release may be mediated significantly by pannexin-1 hemichannels. ***C***. Overall microglial process velocity was similarly decreased with p probenecid (5mM) alone and with concurrent AMPA (1mM) and probenecid (5mM). All significant changes (p<0.05) from baseline are indicated by an asterisk (*).

ATP release in the nervous system, both within [32] and outside the retina [33], [34], have been shown to involve gap junction hemichannels, and in particular a family of large-pore ion channels called pannexins [35]. We hypothesized that ATP release relevant to the regulation of microglial morphology and dynamic properties may also involve this mechanism. We evaluated this by performing recordings before and during the bath application of probenecid (5mM), a selective antagonist of pannexin-1 hemichannels [33], [36], [37]. We found that probenecid exerted a general decrease on all three morphological parameters (Fig 9A). Concurrent application of AMPA in presence of pannexin-1 blockade was unable to increase microglial morphological parameters (Fig. 9B), as was observed when AMPA was applied alone. Overall microglial process velocity was similarly slowed in the presence of probenecid alone, which was similarly not rescued by the concurrent application of AMPA (Fig. 9C). These results indicate that ATP signaling onto microglia is likely to significantly involve the opening of pannexin-1 hemichannels occurring “downstream” in response to ionotropic glutamatergic transmission.

## Discussion

### Functional significance of regulating microglial morphology and process motility

In the basal state, microglia across different regions of the CNS possess a typical branching and ramified morphology that distinguishes them from tissue macrophages outside the CNS [38]. These “resting” microglia in both the brain and the retina have been observed to exhibit a remarkable motility in their processes that is thought to confer properties central to CNS housekeeping functions [1], [2], [3]. The thin, tapering geometry of microglial processes facilitates physical access to narrow intercellular spaces in the densely packed neuropil. Process dynamism also enables microglia to make direct and repeated physical contact with surrounding cells without having to migrate or physically translocate their cell bodies. The extent to which microglia interact with other cell types in the CNS is likely regulated as a function of their morphology and behavior. This regulation is likely to involve non-cell autonomous mechanisms such as local extracellular cues; indeed morphological parameters of individual microglia have been found to vary between different brain regions and between grey and white matter [39]. Factors that induce an increase in dendritic area would have the effect of increasing the volume of neural parenchyma “covered” by each microglial cell, while those that increase in total dendritic process length, branching complexity, and process motility would increase the frequency and density of potential contacts in a microglial cell's “territory”. As rapid and constitutive process dynamism entail energetic and metabolic expenditures, it is probably advantageous for it to be regulated according to the changing needs of the neural parenchyma. Inasmuch as microglial process dynamism is thought to provide synapse maintenance [8], [40] and to deliver trophic factors to neurons [9], it would be consistent to hypothesize that signaling mechanisms that reflect neuronal activity would be well suited to regulate microglial morphology and behavior in this regard.

### Ionotropic neurotransmission as a rapid regulatory signal for microglia

In this study, we have demonstrated that microglia morphology and dynamic behavior are positively regulated by ionotropic glutamatergic neurotransmission and negatively regulated by ionotropic GABAergic neurotransmission. This reciprocal regulation by glutamatergic and GABAergic neurotransmission was found to alter microglial morphology and behavior at a rapid rate, taking effect within a few minutes of neurotransmitter application or blockade. The excitatory and inhibitory natures of ionotropic glutamatergic and GABAergic transmission respectively suggest that these influences converge to determine the overall level of neuronal/synaptic activity which in turn influences microglia dynamism. In a recent study involving microglia in brain slices, it was reported that neither focal electrical stimulation nor local puffs of glutamate or GABA were able to elicit microglial chemotaxis or alter microglial process motility, leading the authors to conclude that microglia may not be sensitive enough to respond to acute neuronal activities [41]. However, in our experiments involving either overall neurotransmitter blockade/stimulation by the bath application of antagonists/agonists, we observed significant alterations in process motility and morphology in response. One explanation for these differences may be that microglial morphology and behavior respond more overtly to overall changes in the level of neurotransmission across the region, rather than to local changes. The indirect mode of regulation through a secondary signal, such as ATP produced by an interconnected network of neurons or macroglia, is also consistent with a broader, rather than local, spatial range of regulation.

Previous studies have reported that microglia are capable of expressing ionotropic neurotransmitter receptors on their cell surfaces [42], and that the activation of these receptors can induce responses such as cytokine expression [27], [29] and chemotaxis [28]. However, these observations were made in studies performed primarily in cultured microglia [27], [28], [29], [43], and were in some cases found in only a minority of microglia [29]. *In vivo*, glutamate receptor expression in microglia was documented only following the induction of ischemic injury [44]. These findings suggest that direct expression of glutamatergic receptors may occur significantly only in microglia that have been activated either in culture or following injury. Consistent with previous electrophysiological studies in brain slices [41], our patch-clamp recordings of microglia in retinal explants did not reveal any direct microglial responses to applications of glutamatergic or GABAergic agonists. We were also unable to co-localize glutamate receptor subunits to the surface of “resting” retinal microglia *in vivo*. Taken together, these findings indicate that glutamate and GABA receptors are likely absent on the surface of “resting” microglia in intact tissue and that the regulatory effects of neurotransmission are mediated indirectly through a secondary signal, likely ATP.

### ATP as an indirect signal for the neurotransmitter-based regulation of resting microglial morphology and motility

The role of ATP in regulating microglial morphology and motility has been previously demonstrated *in vitro* [24], [26], [45], [46] and *in vivo* [1]. Cultured microglia in response to ATP increase membrane ruffling and chemokinesis and migrate along an ATP gradient [24]. Microglia in the intact brain can also project processes toward focal applications of ATP [1]. These ATP effects are mediated directly by P2X and P2Y receptors expressed by microglia [47]. In our patch-clamp recordings of retinal microglia, while a large inward current was elicited with ATP application, no electrical responses were recorded when glutamate or GABAergic agonists are locally applied. These results indicate that “resting” ramified microglia respond directly to ATP and only indirectly to glutamate or GABA neurotransmission.

The regulatory effects of ionotropic glutamatergic and GABAergic transmission thus may regulate ATP release in the retina, which in turn modulates microglial morphology and behavior. Our results show that the application of exogenous ATP was able to induce microglial changes even in the presence of glutamatergic blockade, indicating that ATP release may occur “downstream” to glutamate receptor activation. Consistent with this mechanism, the blockade of P2 purinergic receptors also decreased the effects of AMPA application in microglial cells. As such, ATP signaling onto retinal microglia may indeed be intermediary to ionotropic glutamatergic neurotransmission and may possibly involve astrocytes, Müller cells, and neurons as the potential sources from which ATP release occurs. Our experiments indicated that ATP release onto microglia cells may occur through large conductance pannexin hemichannels. Pannexins have been found to be broadly expressed in different areas of the nervous system [48], [49], including the retina [50], by both astrocytes [51] and neurons [34], [52], although the precise distribution between cell types and subcellular locations remain incompletely understood. Activated by depolarization [53], pannexin hemichannels have been implicated in various forms of non-synaptic communication in neurons and astrocytes, including the modulation of synaptic physiology [54], the propagation of calcium waves [55], and the stimulation of inflammasome, a multiprotein complex involved in innate immunity [56]. However, their involvement in the regulation of microglial morphology and behavior has not been previously described. In our experiments, the decreases in microglial morphology and motility caused by the blockade of pannexin hemichannels was also not rescued by the concurrent application of AMPA, further supporting the notion that ATP release, as well as the reception of ATP signals, occur “downstream” of glutamatergic neurotransmission.

Interestingly, our observations on the role of ionotropic glutamatergic signaling on microglial morphology and motility indicated that the effects of fast AMPA/kainate receptors were considerably larger than those mediated by slower NMDA receptors. The reasons for these differences may potentially relate the regulation of pannexin hemichannels opening in the context of neuron-microglia signaling. It has been noted that the gating and electrophysiological properties of Panx1 channels may vary depending on the mechanisms triggering their activation [35]. Although NMDA receptor stimulation has been demonstrated to trigger pannexin hemichannel opening in pyramidal neurons and drive epileptiform seizure activity [54], the relative contributions of NMDA and non-NMDA receptor signaling to the regulation of pannexin hemichannel gating in the retina remains unclear and constitutes an area of future investigation.

### Communication between neuronal, macroglial, and immune components in the retina

Collectively, our findings indicate that resting microglia morphology and behavior are not controlled solely by cell-autonomous mechanisms but are instead regulated by different forms of neurotransmission. These neurotransmitter effects on microglia are exerted rapidly over a time scale of minutes and involve ionotropic glutamatergic and GABAergic transmission acting in concert to reciprocally regulate microglia behavior. The glutamatergic neurotransmitter effects are likely exerted indirectly via ATP signaling onto microglia as released at least in part by pannexin hemichannels. While the identity of the cells in the retina that release ATP onto microglia remains to be determined, our results lend support to the existence of constitutive and ongoing communication between the neural and immune components of the CNS. The responsiveness of microglial morphology and behavior to neural activity also sheds light on the endogenous function of resting microglia in maintaining homeostasis in the CNS and may lead to insights on how alterations in microglia physiology may play a role in diseases in the brain and the retina.

## Supporting Information

Movie S1 Time-lapse confocal movie consisting of merged Z-stacks of time-lapse confocal images taken of a representative retinal microglia cell in a flat-mounted retinal explant, demonstrating a typical response to the superfusion and subsequent washout of 10 µM NBQX. Frames labeled “baseline” represent images taken under control conditions in which oxygenated Ringer's solution was superfused. Frames labeled “NBQX” represent images taken when the superfusate was replaced with Ringer's solution containing 10 µM NBQX, while those labeled “wash” represent images taken when Ringer's solution was again superfused. Frames in the movie are captured 10 s apart.
(AVI)

Movie S2 Time-lapse confocal movie consisting of merged Z-stacks of time-lapse confocal images taken of a representative retinal microglia cell in a flat-mounted retinal explant, demonstrating a typical response to the superfusion and subsequent washout of 100 µM AMPA. Frames labeled “baseline” represent images taken under control conditions in which oxygenated Ringer's solution was superfused. Frames labeled “AMPA” represent images taken when the superfusate was replaced with Ringer's solution containing 100 µM AMPA, while those labeled “wash” represent images taken when Ringer's solution was again superfused. Frames in the movie are captured 10 s apart.
(AVI)

Movie S3 Time-lapse confocal movie consisting of merged Z-stacks of time-lapse confocal images taken of a representative retinal microglia cell in a flat-mounted retinal explant, demonstrating a typical response to the superfusion and subsequent washout of 150 µM bicuculline. Frames labeled “baseline” represent images taken under control conditions in which oxygenated Ringer's solution was superfused. Frames labeled “Bicuculline” represent images taken when the superfusate was replaced with Ringer's solution containing 150 µM bicuculline, while those labeled “wash” represent images taken when Ringer's solution was again superfused. Frames in the movie are captured 10 s apart.
(AVI)

Movie S4 Time-lapse confocal movie consisting of merged Z-stacks of time-lapse confocal images taken of a representative retinal microglia cell in a flat-mounted retinal explant, demonstrating a typical response to the superfusion and subsequent washout of 1mM ATP. Frames labeled “baseline” represent images taken under control conditions in which oxygenated Ringer's solution was superfused. Frames labeled “ATP” represent images taken when the superfusate was replaced with Ringer's solution containing 1mM ATP, while those labeled “wash” represent images taken when Ringer's solution was again superfused. Frames in the movie are captured 10 s apart.
(AVI)

Movie S5 Time-lapse confocal movie consisting of merged Z-stacks of time-lapse confocal images taken of a representative retinal microglia cell in a flat-mounted retinal explant, demonstrating a typical response to the superfusion and subsequent washout of both 1mM ATP and 10 µM NBQX. Frames labeled “baseline” represent images taken under control conditions in which oxygenated Ringer's solution was superfused. Frames labeled “NBQX + ATP” represent images taken when the superfusate was replaced with Ringer's solution containing 1mM ATP and 10 µM NBQX, while those labeled “wash” represent images taken when Ringer's solution was again superfused. Frames in the movie are captured 10 s apart.
(AVI)

Movie S6 Time-lapse confocal movie consisting of merged Z-stacks of time-lapse confocal images taken of a representative retinal microglia cell in a flat-mounted retinal explant, demonstrating a typical response to the sequential superfusion 10 µM NBQX and then both 1mM ATP and 10 µM NBQX concurrently. Frames labeled “baseline” represent images taken under control conditions in which oxygenated Ringer's solution was superfused. Frames labeled “NBQX” represent images taken when the superfusate was replaced with Ringer's solution containing 10 µM NBQX only, while those labeled “NBQX + ATP” represent images taken when the superfusate was replaced with Ringer's solution containing both 1mM ATP and 10 µM NBQX. Those labeled “wash” represent images taken when Ringer's solution was again superfused. Frames in the movie are captured 10 s apart.
(AVI)
